# Impact Mineralization of Chokeberry and Cranberry Fruit Juices Using a New Functional Additive on the Protection of Bioactive Compounds and Antioxidative Properties

**DOI:** 10.3390/molecules25030659

**Published:** 2020-02-04

**Authors:** Sabina Lachowicz, Jan Oszmiański, Martyna Wilczyńska, Grzegorz Zaguła, Bogdan Saletnik, Czesław Puchalski

**Affiliations:** 1 Department of Fermentation and Cereals Technology, Faculty of Biotechnology and Food Science, Wrocław University of Environmental and Life Science, 37 Chełmońskiego Street, 51-630 Wroclaw, Poland; 2 Department of Fruit, Vegetable and Plant Nutraceutical Technology, Faculty of Biotechnology and Food Science, Wrocław University of Environmental and Life Sciences, 37 Chełmońskiego Street, 51-630 Wrocław, Poland; jan.oszmianski@upwr.edu.pl (J.O.); martyna28b@gmail.com (M.W.); 3 Department of Bioenergetics and Food Analysis, Faculty of Biology and Agriculture, University of Rzeszów, 35-601 Rzeszów, Poland; g_zagula@univ.rzeszow.pl (G.Z.); bogdan.saletnik@ur.edu.pl (B.S); cpuchal@ur.edu.pl (C.P.)

**Keywords:** chokeberry, cranberry, chicken eggshell powder, antioxidative activity, polyphenol compounds, mineralization

## Abstract

Chicken eggshells can be used as an attractive dietary source of mineral compounds, including calcium (Ca). However, the effects of chicken eggshell powder (CESP) on berry fruit juices have not been studied to date. Therefore, the objective of this study was to evaluate the effect of its addition to juices from chokeberry and cranberry on their phytochemical properties. The juices were determined for contents of polyphenols (determined by ultra-efficient liquid chromatography coupled with a mass detector (UPLC-PDA-ESI-MS/MS)), macro- and microelements (by inductively coupled plasma - optical emission spectrometry (ICP-OES)), and organic acids (by high-performance liquid chromatography (HPLC-PDA)) as well as for their antioxidative activity by radical scavenging capacity (ABTS) and ferric reducing antioxidative power (FRAP) assay, color profile (CIE L* a* b* system), and sensory attributes. The study results demonstrate that CESP addition to chokeberry and cranberry juices enriched them with minerals and increased their Ca content 25.7 times and 66.3 times, respectively, compared to the control samples. Juices supplementation with CESP significantly decreased their acidity and total organic acids content as well as increased their pH value. Chokeberry and cranberry juices supplementation with 1% CESP caused no significant changes in the amount of precipitate and their color, but it significantly improved their taste. For this reason, CESP addition in the amount of up to 1% can be suggested as the optimal supplementation of berry fruit juices. The study also demonstrated that CESP addition in the amount of up to 1% caused no significant differences in the content of polyphenolic compounds and in the antioxidative activity of juices, which can be deemed important from the viewpoint of their putative health benefits. In addition, the heat treatment of juices contributed to only a 4% loss of polyphenolic compounds from the CESP-supplemented juices compared to the 6% loss from the non-supplemented juices.

## 1. Introduction

Functional-type foods are foods that exert a positive impact on human health and well-being and also reduce the risk of development of various diseases due to the bioactive components with which they had been enriched [1]. The functional food sector is the youngest but the most prospective and developing branch of the food industry. In 2000, the value of the global functional food market was estimated at 30 billion USD. In 2008, it increased to 80 billion USD and in 2018 it increased to 160 billion USD; and it is estimated to reach 253 billion USD by the year 2024 [2]. This boost has been attributable to the successively growing interest of consumers in healthy nutrition, organic food, functional food, and food enriched with natural components, including mainly those that exhibit antioxidative potential [3]. 

The natural bioactive compounds of food exert a positive impact on human health by affecting a variety of physiological and cellular functions in the body. Their significant amounts can be found in fruit and vegetables, whose consumptions in the fresh or little-processed form yields some health benefits such as, e.g., delay of the aging process or reduction of the risk of inflammatory conditions development. A lack or insufficient amounts of fruit and vegetable in an everyday diet can underlie the development of such diseases as: atherosclerosis, diabetes, cancers, and those related to the cardiovascular system [4,5]. Hence, one of the possibilities to produce functional foods is to use berry fruits, such as e.g., chokeberry or cranberry, which are exceptionally rich in bioactive polyphenols that exhibit antioxidative activity. Food products made of these fruits can additionally be enriched with other substances, including e.g., mineral compounds, because previous studies have demonstrated a serious problem posed by the insufficient supply of calcium in the diet compared to the recommended nutritional standards [6]. Today, mainly calcium carbonate (CaCO_3_) is used as the major source of calcium in the production of dietary supplements, due to a high content of Ca^2+^ ions (40%) [7]. Synthetic calcium carbonate has a low bioavailability; thus, its natural sources are a focus of interest among scientists. Recently, preparations made of the external skeletons of marine organisms have become increasingly popular. However, this source of calcium has many drawbacks, such as the relatively high cost of production, limited availability, and the risk of accumulation of such heavy metals as mercury, cadmium, and lead [8].

Ample research has shown eggshells to be a fine alternative source of highly bioavailable macro- and microelements, such as calcium. In addition to their high content of calcium, eggshells are composed of many vital microelements including strontium, which has a positive effect on bone metabolism [8,9,10,11]. One whole medium-sized eggshell makes about one teaspoon of powder, which yields about 750–800 mgs of elemental calcium plus other microelements [11]. The intake of eggshell-derived calcium has been proved to positively affect bone density and to exert anti-rickets effect, while in osteoporotic patients, it strengthens bones by calcium resorption. The composition of an eggshell is very similar to that of our bones and teeth. Patients suffering from osteoporosis are recommended to take 400–500 mg Ca per day to supplement its dietary sources. In addition, the pharmaceutical industry uses eggshells as a valuable source of calcium with an extraordinarily high bioavailability: 90% [9,10,11]. 

Food enrichment with chicken eggshells and other organic sources of calcium is still little known. Studies conducted so far have addressed the impact of using chicken eggshell powder as a source of calcium on biscuits, chocolate cake, and wheat breads. Hassan [12] demonstrated a significantly higher content of calcium in the produced biscuits that reached 607, 1378, and 2175 mg/100 g, respectively. According to Ray et al. [13], the powdered eggshells also had a positive impact on the calcium content increase in chocolate cake, whereas Bradauskiene et al. [14] confirmed their positive effect on the final calcium content in wheat breads that were characterized by a better appearance of the skin, higher overall acceptability, and showed no significant changes in their taste and aroma [14]. Considering the above, it seems worthwhile to extend the applicability of eggshells in the food industry, especially in the sector of beverages. Being soluble in sour juices from chokeberry and cranberry, the ions of calcium and other minerals of chicken eggshell powder (CESP) can be better absorbed due to their organic derivatives. There is much evidence that the extent of calcium absorption from the gastrointestinal tract is largely determined by the solubility of calcium preparation in the stomach. Calcium carbonate of natural origin was found to be far more soluble than the synthetic CaCO_3_. According to these authors, the chicken eggshell powder exhibited a significantly higher solubility due to its porous structure [15].

One of the possible ways to enrich chokeberry and cranberry juices in calcium-rich products is their mineralization. This study aimed to evaluate the applicability of chicken eggshells as an excellent source of minerals. Therefore, its objective was to evaluate the effect of the addition of chicken eggshell powder (CESP) to berry fruit juices from chokeberry and cranberry on their physicochemical properties. The juices were determined for contents of polyphenolic compounds (determined by ultra-efficient liquid chromatography coupled with a mass detector (UPLC-PDA-ESI-MS/MS)), macro- and microelements (by inductively coupled plasma - optical emission spectrometry (ICP-OES)), and organic acids (by high-performance liquid chromatography (HPLC-PDA)) as well as for their antioxidative activity (by radical scavenging capacity (ABTS) and ferric reducing antioxidative power (FRAP) assay) and color profile (CIE L* a* b* system). They were also subjected to the sensory evaluation. To the best of our knowledge, the effects of chicken eggshell powder on berry fruit juices have not been investigated so far.

## 2. Results and Discussion

### 2.1. Determination of Basic Physicochemical Parameters

**Figure 1:**
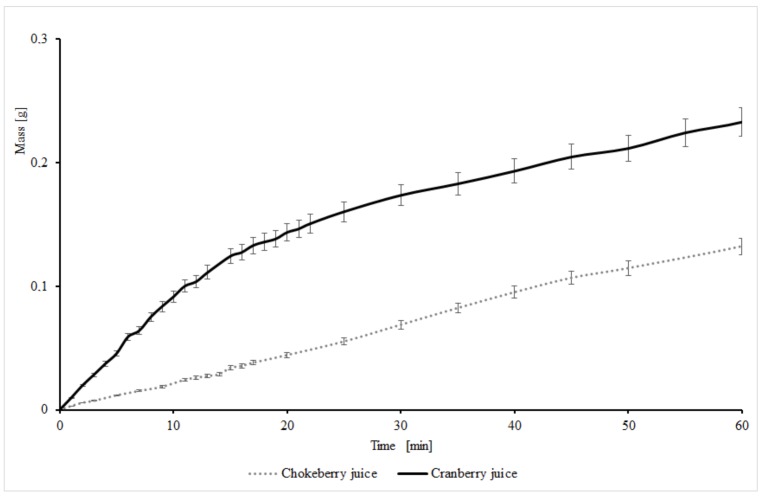
Effect of weight change in juices as a result of carbon dioxide evolution in reaction with eggshells.

Figure 1 depicts the course of changes in juice weight due to the reaction with CESP. The foaming of the samples was observed as a result of gas emission. The highest intensity of CESP dissolution was determined for the samples of cranberry juice, because they were characterized by the most intense foaming and weight loss during the reaction. These samples revealed also the fastest and the most intense gas emission. A lesser intensity of foaming and gas emission was observed for chokeberry juice samples. The process of gaseous products’ liberation can be due to the course of the reaction between berry fruit juices and chicken eggshell powder. After 60 min, weight loss from the same amount of the mixture of juice and CESP reached 0.45% and 0.26% for cranberry and chokeberry juice, respectively. Based on the analysis of the chemical composition of both eggshells and fruits, it may be speculated that it was carbon dioxide that diffused from the mixture. It is probably produced due to the inhibition of the exchange reaction. A weak salt (calcium carbonate) of the powder degrades to carbon dioxide and calcium ions. Carbon dioxide is the by-product of this reaction; it can be noticed as bubbles liberating from the mixture (Figure 1).

**Figure 2:**
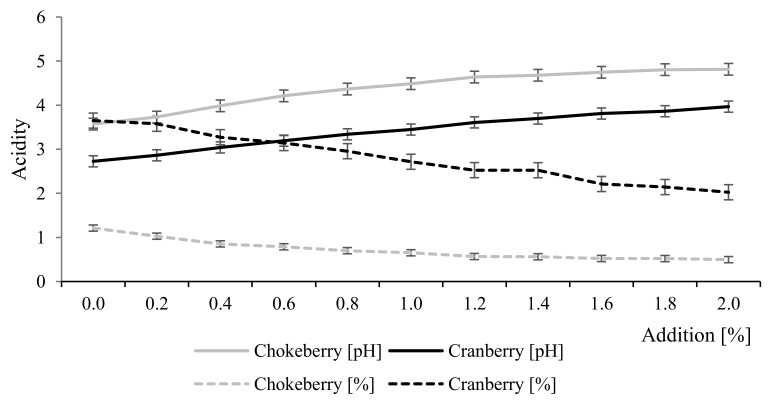
The effect of the addition of eggshells on acidity changes in berry fruit juices before thermal treatment.

Figure 2 and Table 1 present the changes in total acidity, active acidity (pH), and color parameters (CIE L*, a*, b*) of chokeberry and cranberry juices caused by the dissolution of selected doses of CESP. Statistically significant changes were observed in the physicochemical parameters of juices depending on juice type and CESP dose. Carbon dioxide emission increased along with an increasing dose of CESP additive. Heat treatment had no significant effect on the values of the analyzed parameters. In turn, depending on juice acidity, the addition of chicken eggshell powder to chokeberry and cranberry juices caused a significant decrease in their total acidity and an increase in their active acidity. The higher the dose of the additive, the lower the value of total acidity and the higher the pH value. The 2% addition of CESP contributed to a significant reduction in the total acidity of juices, i.e., by ca. 49% and 45% in chokeberry and cranberry juice, respectively. In the case of active acidity, the pH value increased after 2% addition of CESP by 26% and 31% chokeberry and cranberry juice, respectively. The initial total acidity and active acidity of the non-supplemented juices were consistent with literature data [16,17,18]. The results obtained suggest that CESP addition contributed to the neutralization of organic acids of berry fruit juices. Hence, the maximum dose of the powder additive can be up to 1% for chokeberry juice and up to 2% for cranberry juice. The pH values of juice higher than 4.5 could necessitate the sterilization process aimed at preserving the finished products. In turn, the process of sterilization could contribute to greater losses of biologically active components compared to the pasteurization process [19,20]. Similar observations were made for breads enriched with CESP. Their total acidity decreased 2.0 times, whereas their pH value increased 1.4 times compared to the non-supplemented breads [14]. The juices were also determined for their organic acid profile, using HPLC-PDA (Table 2). The results obtained indicate that the heat treatment of juices had no significant effect on the contents of their organic acids; however, these were affected by juice type and additive dose. The six organic acids identified in chokeberry juice included malic (ca. 74.6) > quinic (ca. 15.5%) > isocitric (ca. 4.7%) > fumaric (ca. 3.3%) > citric and oxalic (<1%) acids, whereas the six organic acids identified in cranberry juice included citric (ca. 48.6%) > malic (ca. 34.8%) > succinic (ca. 12.3%) > isocitric (ca. 3.3%) > galacturonic and oxalic (<1%) acids. Malic acid was found to be the major organic acid of chokeberry juice, whereas citric acid was the major organic acid of cranberry juice, which is consistent with findings reported by other authors [21,22]. The addition of chicken eggshell powder to cranberry juice was demonstrated to insignificantly decrease the content of its organic acids, i.e., by 3% on average. In the case of chokeberry juice, it decreased organic acids content by 10% on average. The greatest losses were observed for isocitric and quinic acids (by 38% and 27%, respectively). The CESP addition at 1% had a significant effect on contents of the analyzed acids, whereas its successive doses caused insignificant differences. Changes in the acidity of the examined samples after their supplementation with CESP were due to their neutralization with metal ions [14].

Another analyzed parameter of the juices was their color, which was evaluated in the CIE L*, a*, b* system. The type of juice, its heat treatment, and its supplementation with CESP were found to contribute to significant changes in its color parameters. The results of color analysis demonstrate that the non-heated chokeberry juice darkened (a decrease in L* value) along with increasing CESP addition. The color of chokeberry juices turned from red to dark purple and was dependent on the pH value of juice. Anthocyanins of berry fruit juices turn red in the acidic environment, whereas their color tends to blue in the less acidic medium. In turn, the heat treatment of chokeberry juice contributed to the lighter color of the blank sample compared to the non-heated sample. The color became darker along with increasing powder addition. Greater changes were observed for parameter b* than a* also depending on the pH value of juice. Different dependencies were observed for cranberry juice samples. Regardless of the heat treatment, their color became significantly lighter, whereas the values of the a* and b* parameters decreased depending on the additive dose and indicated color change toward green and blue. The observed changes were positively correlated with the pH value and total acidity of the juices.

### 2.2. Determination of Ash and Mineral Compounds 

**Figure 3:**
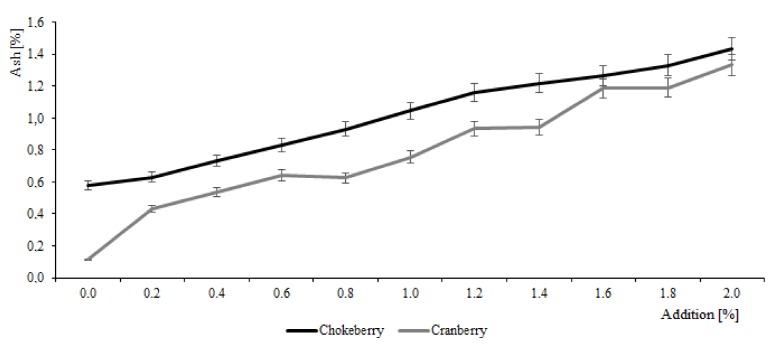
Effect of eggshell addition on ash changes in berry juices before thermal treatment.

Analyses were also conducted to evaluate the effect of berry fruit juices supplementation with CESP on their ash content. Juice type and powder dose had a significant effect on total ash content, which was 4.8 times higher in the chokeberry juice than in the cranberry juice (Figure 3). This has also been confirmed by other authors [17,20]. The chicken eggshell powder represents also a rich source of mineral compounds; hence, its use as a food additive to, e.g., berry fruit juices, is expected to increase the total minerals content of the supplemented products [12,13,23]. The present study showed that the total content of minerals increased in the analyzed juices along with CESP dose increase. Similar observations were made in biscuits and chocolate cakes supplemented with CESP [12,13]. 

**Figure 4:**
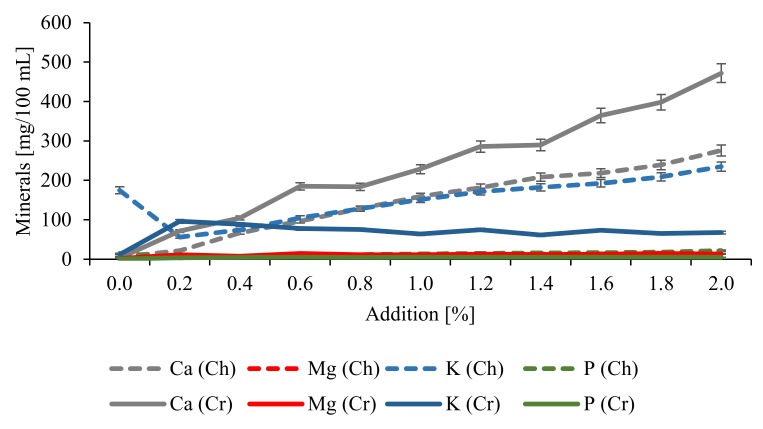
Effect of eggshell addition on mineral compounds changes in berry juices before thermal treatment. *Ch, chokeberry; Cr, cranberry.

Furthermore, analyses were carried out to establish the effect of CESP addition to chokeberry and cranberry juices on the contents of minerals determined with the ICP-OES method (Table 3, Figure 4). The major element of the CESP was calcium, which accounted for 27.70% of the total eggshell weight, as confirmed by Siulapwa et al. [23]. The second element in terms of quantity was magnesium, which accounted for 0.35%, whereas sodium, potassium, phosphorus, sulfur, and strontium accounted for 0.10% to 0.05% of the total eggshell weight. These results indicate that 1 g of CESP can cover 28% of the RDA for calcium (Recommended Dietary Allowance) [24]. According to findings reported by Schaafsma et al. [25], the calcium content in CESP from the Netherlands ranged from 385 to 401 mg Ca/g powder. Differences in calcium content between eggshells are natural and can be due to the age of the layer hen, calcium content in layer bones, feed mixture administered, and many other factors affecting the biomineralization process [12,13,25]. The berry fruit juices were found to contain four major elements: calcium, magnesium, phosphorus, and potassium. Their contents were significantly dependent on juice type and CESP dose (Figure 4), but not on the thermal heating of juices, and they were 5.8 times higher in the chokeberry than in the cranberry juice. CESP addition at 1% contributed to the enrichment of juices with Ca, Mg, K, and P, and their contents were 25.7, 1.2, 2.0, and 1.2 times higher in the chokeberry juice as well as 66.3, 6.2, 5.1, and 4.9 times higher in the cranberry juice, respectively, compared to the control samples. The 2% dose of CESP additionally increased contents of these minerals by 42%, 39%, 36%, and 39% in the chokeberry juice and by 52%, 13%, 6%, and 2% in the cranberry juice. The contents of phosphorus and magnesium increased only to a little extent along with the CESP dose increase because their contents in the eggshell are relatively low, whereas the greatest increase was demonstrated in the case of calcium ions (Figure 4). Similar results were achieved upon CESP addition to biscuits, chocolate cakes, and butter cakes [12,13,26]. 

### 2.3. Determination of Polyphenolic Compounds

**Figure 5:**
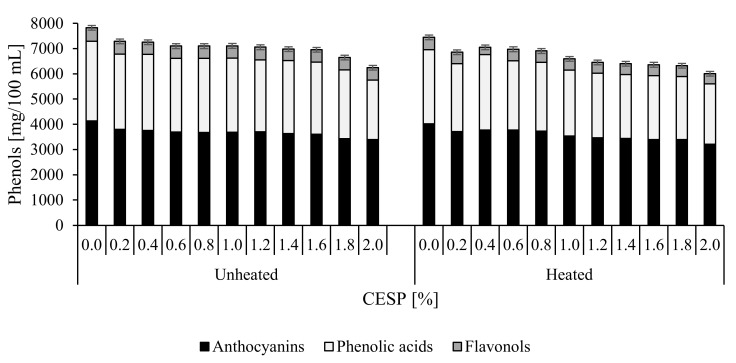
Polyphenolic content in the chokeberry juice with CESP before and after thermal treatment.

**Figure 6:**
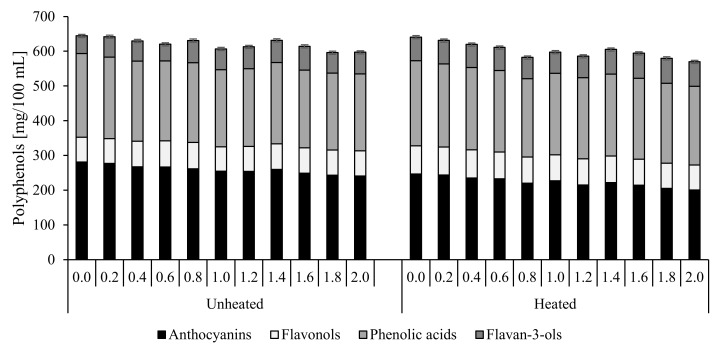
Polyphenolic content in the cranberry juice with CESP before and after thermal treatment.

The identification of 49 compounds, including 21 compounds in cranberry juice and 23 compounds in chokeberry juice, representing anthocyanins, phenolic acids, flavonols, flavones, and flavan-3-ols, was carried out using available standards and literature data as well as data achieved from MS and MS/MS analyses. The respective results are presented in Figure 5 and Figure 6 and in Table 4, Tables S1 and S2. The structure of the identified compounds was already determined in some other studies [17,20,27]. 

To the best of our knowledge and according to the available literature, no research have been conducted so far into the effect of CESP on contents of polyphenolic compounds in non-heated and heated juices from chokeberry and cranberry. In the present study, we tried to establish whether the CESP addition may contribute to losses of polyphenols in juices being exceptionally rich in these compounds. This is especially important from the viewpoint of health benefits offered by their consumption. The total content of polyphenolic compounds in the analyzed juice samples depended on juice type, its thermal treatment, and CESP dose added. Analytic results (Figure 5 and Figure 6) demonstrate the chokeberry juice to be a richer source of polyphenols; their content was ca. 15 times higher than in the cranberry juice, which is in agreement with literature data [28]. Juice supplementation with increasing doses of the chicken eggshell powder (from 0.2% to 2%) increased the total losses of polyphenolic compounds from 6.8% to 20.2% in chokeberry juice and from 2.4% to 9.1% in cranberry juice. The greater losses of polyphenols noted along with an increasing dose of CESP were probably due to their sedimentation with the powder [29]. Therefore, while planning juice supplementation with CESP, its doses should be carefully considered to minimize losses of polyphenols. The recommended dose of the additive is ca. 1%, which allows minimizing their losses to 9.1% and 7.7% in chokeberry and cranberry juice, respectively. The heat treatment of the control samples caused the loss of polyphenolic compounds by 4.8% in the chokeberry juice and by 2.5% in the cranberry juice. The addition of CESP contributed to negligible losses of polyphenols in the heat-treated juices compared to the control samples. On average, the total losses of polyphenols noted for all doses of the additive reached 5.4% in the chokeberry juice and 3.4% in the cranberry juice. Pasteurization can be applied to juices supplemented with CESP additives, as it does not impair juices’ enrichment with minerals and ensures the preservation of their biologically active polyphenolic compounds. 

The major polyphenolic compounds identified in juices included anthocyanins > phenolic acids >> flavanols > flavan-3-ols. Cranberry juices were found to contain eight anthocyanins, including derivatives of cynidin, delfinidin, malvidin, and peonidin, which accounted for 42% of the total polyphenols on average, whereas eight anthocyanin derivatives were identified in chokeberry juices that represented 54% of the total polyphenols (Figure 5 and Figure 6). The major identified compounds were peonidin-3-*O*-galactoside in the cranberry juice and cyanidin-3-*O*-galactoside in the chokeberry juice. The anthocyanins content in the cranberry juice was 14.7 times lower than in the chokeberry juice, which is consistent with literature data [17]. In chokeberry juices supplemented with CESP doses increasing from 0.2% to 2%, it decreased by 8% to 17.9%, and this difference was statistically significant compared to the control sample. However, in the samples supplemented with CESP doses from 0.2% to 1%, anthocyanins losses were small and increased only from 8% to 10.8%, whereas the higher CESP doses (1.8% and 2%) contributed to over 17% loss of anthocyanins, and this difference was statistically significant. In cranberry juices supplemented with CESP, the losses of anthocyanins compared to the control sample were smaller than in the chokeberry juices. They ranged from 1.4% to 14% at additive doses increasing from 0.2% to 2%, and this difference was statistically significant. However, in the samples supplemented with additive doses from 0.2% to 0.8%, these losses were negligible and increased only from 1.4% to 6.9%.

The heat treatment of juices contributed to 2.8% losses of these compounds in the control sample. In turn, the use of the CESP additive had a significant effect on the content of anthocyanins in heat-treated chokeberry juices; in the juice with the highest (2%) addition of CESP, the losses of anthocyanins reached only 5.5% compared to the non-heated sample. An opposite dependency was determined in the cranberry juice, in which the compounds turned out to be less stable. In the control sample, the loss of anthocyanins caused by the heat treatment reached 12.3%, compared to the loss of 16.6% determined in the sample with the highest CESP addition (2%). 

Phenolic acids represent another group of chokeberry and cranberry polyphenols. The liquid chromatography method employed allowed identifying eight phenolic acids in cranberry juice and four in chokeberry juice. Chlorogenic acid was found to be the major acid in all samples. Its concentration is very important, considering its role as a precursor of the taste of fruit-based food products [30]. The evaluation of the effect of CESP addition to juices demonstrated that losses of phenolic acids in chokeberry juice ranged from 5.5% in the sample with the lowest CESP dose to 25.4% in that with the highest CESP dose. These analyses point to the reaction of phenolic acids with metals of CESP and their precipitation from juice. Hence, it is more advisable to use smaller doses of CESP, e.g., 1%, because losses of phenolic acids in the juice supplemented with this dose of the additive reached only 7%. Losses of phenolic acids in the cranberry juice were smaller and ranged from 2.6% in the sample with the lowest CESP dose to 8% in that with the highest dose of the additive. The heat treatment of juices had no significant effect on losses of phenolic acids, which are more resistant to heating than anthocyanins [29].

Flavonols represent another, the smallest, group of chokeberry and cranberry polyphenols. The ultra-efficient liquid chromatography coupled with a mass detector (UPLC-PDA-ESI-MS/MS technique) allowed identifying 20 flavonol derivatives of quercetin, myricetin, isorhamnetin, syringetin, and methoxyquercetin, including 11 flavonol glycosides identified in cranberry juice and 12 identified in chokeberry juice. Flavonol derivatives are important to human health as they are effective antioxidants [31]. In general, they exhibit a higher antioxidative activity compared to anthocyanins having the same hydroxylation formulas, as determined with the oxygen radical absorbance capacity (ORAC) method [28]. The addition of CESP caused no significant differences in the content of flavonols in the chokeberry juice; the difference noted between the chokeberry juice with the lowest CESP dose and the control sample reached 5%, whereas it was 8% between the chokeberry juice with the highest CESP dose and the control sample. In the cranberry juice, no losses of flavonols were demonstrated upon CESP addition. However, their content was significantly affected by the thermal treatment of juices. In the chokeberry juices, losses of flavonols ranged from 9.4% in the juice with the lowest CESP dose to 17.8% in that with the highest CESP dose. These differences can probably be attributable to differences in the structure of juice flavonols [32]. 

The last identified group of compounds were the flavan-3-ols, which were detected only in the cranberry juice as derivatives of (+)catechin and (−)epicatechin. Their contents were comparable with literature data [17]. The heat treatment of juices and CESP addition contributed to a significant increase in their content; they were probably released from procyanidins having a higher degree of polymerization to the compounds with a lower mass of flavan-3-ols. Literature data also confirm the impact of temperature on the increased concentrations of these compounds in berry fruit juices [33].

### 2.4. Determination of Antioxidant Activity

The antioxidative activity of the analyzed juices without and with the addition of CESP (heated and non-heated) was measured with the radical scavenging capacity) (ABTS) and ferric reducing antioxidative power (FRAP) methods, and the respective results are presented in Table 5. The effect of CESP addition to fruit juices on their antioxidative activity has not been studied before. The antioxidative activity of the non-supplemented chokeberry juice reached 10.94 and 9.79 mM TE/mL when assayed with the ABTS and FRAP tests, respectively, and it was 7.9 and 6.5 times higher than in the cranberry juice. The antioxidative activity of the analyzed juices was similar to that reported earlier by Lachowicz et al. [29] and Oszmiański et al. [17]. The results achieved in the present study showed that powder addition and juice type had a significant effect on the antioxidative potential of juices. Before the heat treatment, the mean antioxidative activity of the chokeberry juice with CESP addition was lower by 36% and 39%, and that of the cranberry juice was lower by 25% and 21% compared to the control samples when assayed with the ABTS and FRAP tests, respectively. In the heated samples, the differences compared to the control samples were similar, whereas CESP addition did not suppress the antioxidative activity during the heat treatment. 

### 2.5. Determination of Sensory Evaluation

**Figure 7:**
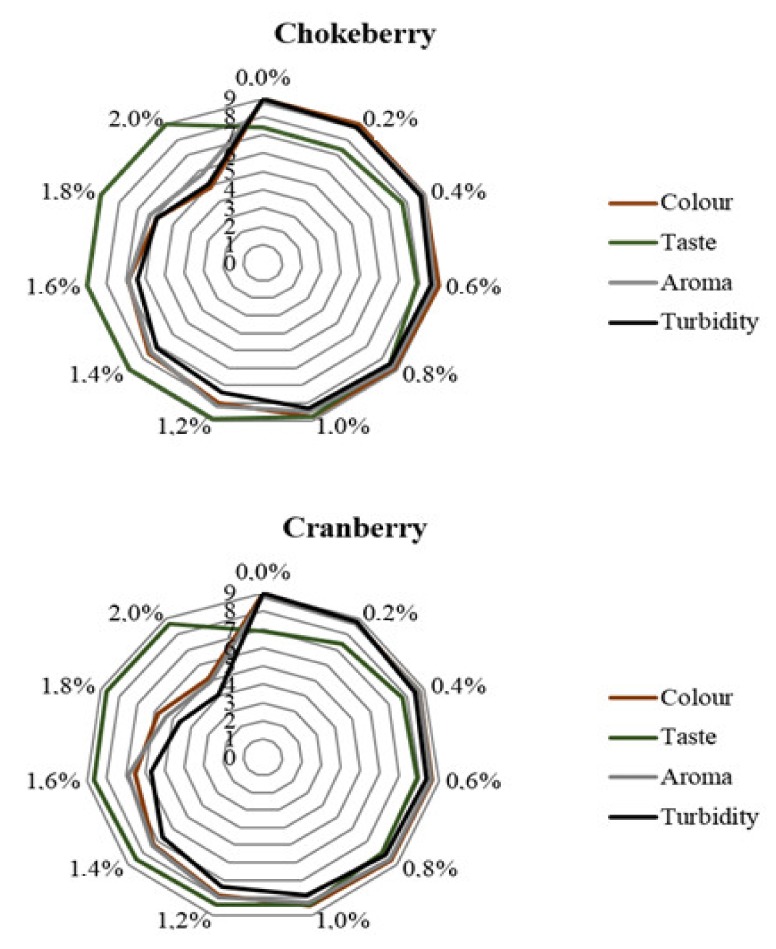
Sensory analysis in products with CESP before thermal treatment.

The juices were also subjected to the sensory evaluation to assess their taste (with attention paid to the sour taste), aroma, color, and sediment precipitation. The heat treatment had no significant effect on the sensory attributes of juices; hence, the results are presented only for the non-heated samples (Figure 7). Generally, the results achieved demonstrate that the color and aroma of the analyzed juices deteriorated along with increasing CESP doses. The panelists noted the presence of the sediment, the amount of which depended on CESP dose. According to the scores given by the panelists for these sensory attributes, the maximal dose of the CESP additive should reach 1%. In turn, chokeberry and cranberry juices supplementation with CESP had a positive impact on their taste, as it allowed masking their sour taste. In the case of this sensory attribute, the higher the dose of the additive, the higher the scores given to juices. This was also confirmed by the results of analyses of the total acidity of juices. As reported by Lachowicz et al. [27], taste and color play a significant role in the sensory assessment of foods. For this reason and considering all sensory attributes, the maximal dose of CESP addition to berry fruit juices should not be higher than 1%. According to Bradauskiene et al. [14], CESP addition to breads contributed to significant deterioration of their taste, and its increasing doses caused perceptible granularity during chewing. These authors established 5 g of the powder to be the optimal dose in bread making [14]. In the case of biscuits or chocolate cakes, the optimal CESP addition deemed acceptable by consumers was established at 6% [12,13], because its higher doses contributed to the appearance of fishy odor [34]. In turn, Brun et al. [35] reported that the best form of using chicken eggshells as a dietary supplement of Ca is to add them in the powdered form to, e.g., bread, pizza, and spaghetti, as it caused little changes in their texture and no changes in their taste. Calcium-enriched foods should have similar physical and sensory properties to their non-enriched counterparts [13,36,37,38].

### 2.6. Principal Components Analysis

**Figure 8:**
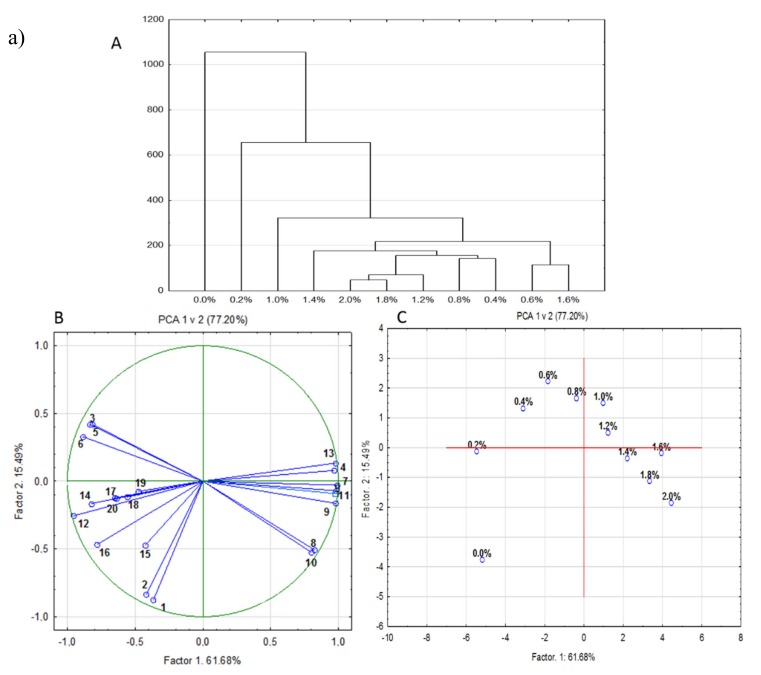

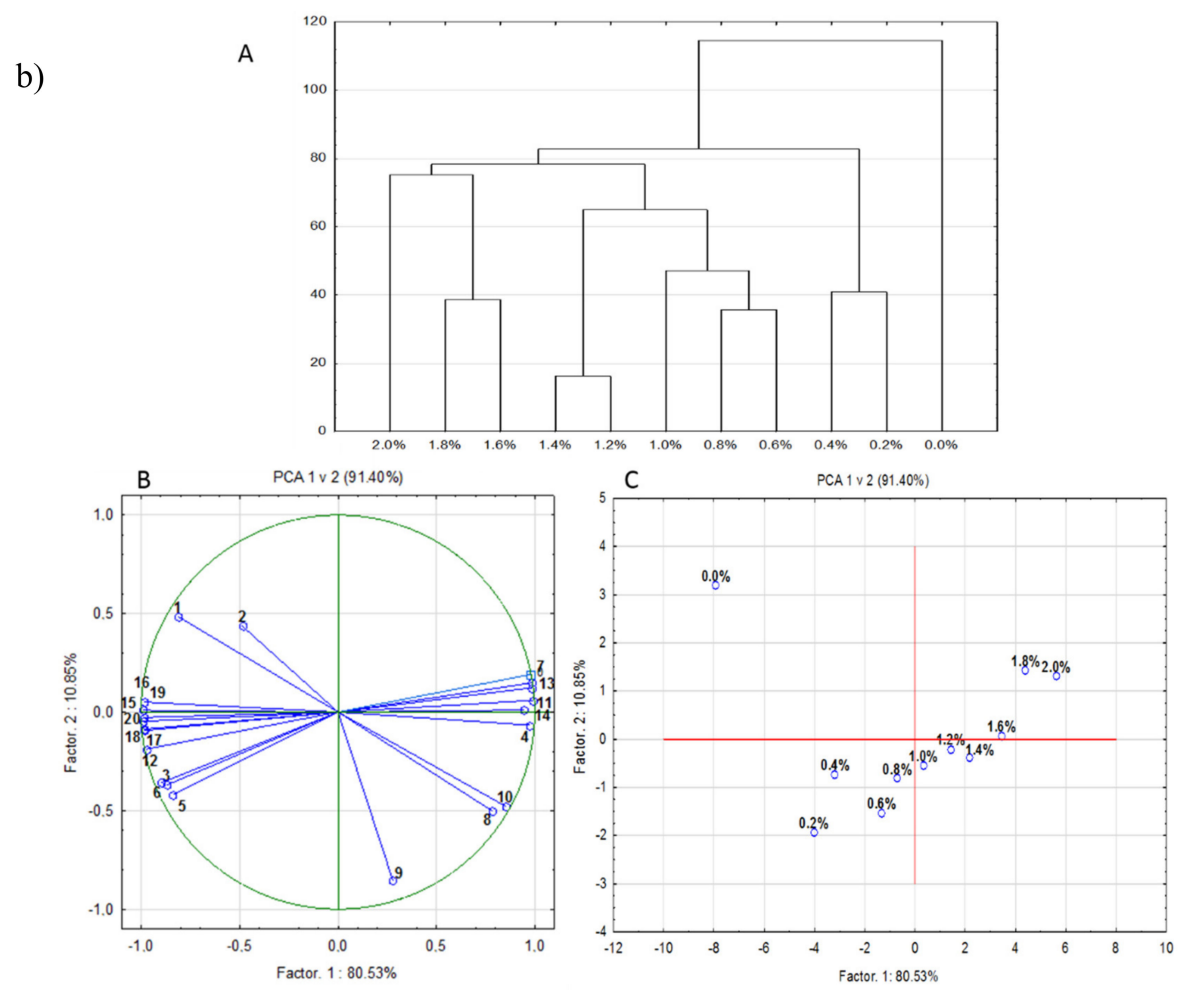
The statistical analysis for chokeberry fruit (**a**) and for cranberry fruit (**b**): (**A**) dendrogram, (**B**) decomposition of measured parameters (1-ABTS; 2-FRAP; 3-Color; 4-Taste; 5-Aroma; 6-Turbidity; 7-Calcium; 8-Magnesium; 9-Potasium; 10-Phosphorus; 11-Ash; 12-Acidity; 13-pH; 14-L*; 15-a*; 16-b*; 17-Anthocyanins; 18-Phenolic acids; 19-Flavonoids; 20-Sum of polyphenolic compounds), (**C**) decomposition of measured samples (from 0.0% to 2.0% of CESP).

The principal component analysis and cluster analysis were used to demonstrate a correlation between the physicochemical parameters of chokeberry and cranberry juices without and with the addition of CESP (Figure 8a and b (A–C)). The cluster analysis distinguished the three classes of measured juices samples without and with CESP. The first group was characterized by the highest antioxidative properties and polyphenolic compounds content (juices without CESP; 0.0% of CESP). The second group was characterized with high antioxidative activity in the sample with CESP (sample with 0.2% and 0.4% of CESP). The last group can be divided into two subclasses. One of the subclasses comprises the juices with CESP with the highest amount of mineral compounds and pH, and the lowest acidity, antioxidative activity, and polyphenolic compounds (sample with 1.6%, 1.8%, and 2.0% of CESP) (Figure 8 a and b (A)).

Figure 8 a and b (B) present the dependence between the major compounds and input variables. This graph illustrates the distribution of the individual measured parameters on the plane for chokeberry (Figure 8a) and cranberry juice (Figure 8b). The principal component analysis explained respectively 77.20% and 91.40% of the total variability for chokeberry juice for cranberry juice, including intergroup variances of PC1 = 61.68% and PC2 = 15.49% and PC1 = 80.53%, and PC2 = 10.85%, respectively. When characterizing the distribution of variables, they may be shared into two groups. PC1 concerned with components related to the amount of mineral compounds, ash, pH, and taste. PC2 focused on components related to antioxidative activity, polyphenolic compounds, and sensorial attributes. In addition, a negative correlation between the parameters of PC1 and PC2 was observed. It can be due to a positive correlation between the values of parameters on the same factors.

Figure 8 a and b (C) present the projection of juices without and with different doses of CESP. The characteristics of the interrelationship between both factors indicate that closely located points indicate their similarity with respect to physicochemical and sensorial composition. Juices without additives were classified into a separate group.

## 3. Materials and Methods 

### 3.1. Reagent and Standard

The compounds 2,2′-azinobis(3-ethylbenzothiazoline-6-sulfonic acid) (ABTS), 6-hydroxy-2,5,7,8-tetramethylchroman-2-carboxylic acid (Trolox), 2,4,6-tri(2-pyridyl)-*s*-triazine (TPTZ), methanol, malic acid, oxalic acid, citric acid, isocitric acid, quinic acid, ferulic acid, galacturonic acid, and succinic acid were purchased from Sigma-Aldrich (Steinheim, Germany). On the other hand, (−)-epicatechin, (+)-catechin, procyanidin B2, chlorogenic acid, neochlorogenic acid, cryptochlorogenic acid, caffeic acid, dicaffeoylquinic acid, *p*-coumaric acid, myricetin, isoquercitrin, cyanidin-3-*O*-galactoside, and cyanidin-3-*O*-glucoside were purchased from Extrasynthese (Lyon, France). Acetonitrile for ultra-performance liquid chromatography (UPLC; Gradient grade) and ascorbic acid were from Merck (Darmstadt, Germany). 

### 3.2. Materials 

The juices made of ‘Galicjanka’ cultivar of chokeberry and ‘Stevens’ cultivar of cranberry used in this study were obtained from a horticulture farm in Wojciechow near Lublin, Poland (51°14′08″N 22°14′41″E) (BioGrim company). To prepare powders from eggshells, contents of the egg were separated, and the shells were washed and cooked in deionized water for 30 min. Once dried, they were ground in a laboratory grinder (IKAA.11.Germany), and the powder obtained was sifted through a screen with mesh size of 0.2 mm. The dry powders were kept until addition to juices. 

### 3.3. Technology

In total, 22 variants of samples were prepared for each of the juices tested. Before adding chicken crust, it was washed twice and boiled in deionized water for 30 min according to the Hassan report [12]. Free-range hens from ‘Rosa’ hens were used for the research. From the same batch, 11 portions of juices (50 g) were weighed into beakers; then, chicken eggshell powder (CESP) was added in successive doses of 0, 0.1, 0.2, 0.3, 0.4, 0.5, 0.6, 0.7, 0.8, 0.9, and 1.0 g at a temperature of 20 °C under continuous stirring for 90 min. This allowed preparing 11 successive samples that were next heated in a water bath at a temperature of 90 °C for 10 min. After cooling, the samples were centrifuged and subjected to analyses. To determine reaction kinetics, additional samples of juices (50 g) with 0.5 g CESP addition were prepared. They were placed on an analytical scale, and their weight loss was monitored for 60 min. Experiments were performed in three replications.

### 3.4. Determination of Ash and pH

The ash [%] and pH were marked in accordance with the Polish Norm PN-EN 1132:1999 and PN-EN 1135:1999.

### 3.5. Determination of Color

The color properties (L*, a*, b*) of chokeberry and cranberry fruit juices were determined by reflectance measurements with a Color Quest XE Hunter Lab colorimeter. The samples were determined according to the method described by Lachowicz et al. [27]. The data were the means of three measurements.

### 3.6. Determination of Mineral Content by ICP-OES Analysis

The composition of chemical elements in the infusions was determined using inductively coupled plasma - optical emission spectrometry (ICP-OES) apparatus (Schaumburg, IL, USA), Thermo iCAP Dual 6500 with horizontal plasma, and capacity for detection along and across the plasma flame (radial and axial). Before measuring each batch of 10 samples, calibration was performed using certified (Merck) models with concentrations of 10,000 ppm for Ca, Mg, K, and P; and 1000 ppm for Al, Cu, S, and Zn.

In each case, a three-point calibration curve was used for each element, with optical adjustment applying the method of internal models, in the form of yttrium and ytterbium ions, at concentrations of 2 mg/L and 5 mg/L, respectively. The analytical methods were validated with two independent tests. In order to identify the relevant measurement lines and avoid possible interferences, the method of adding a model with known concentration was applied (Environmental analysis, Method 200.7, US EPA, Drinking water).

### 3.7. Determination of Organic Acids

Organic acids were determined by HPLC–PDA as described previously by Lachowicz et al. [16]. All data were obtained in triplicate. Results were expressed as mg/L of chokeberry and cranberry fruit juices.

### 3.8. Determination of Polyphenolic Compounds

All analyses of polyphenols of the chokeberry and cranberry fruit juices were carried out using an ACQUITY Ultra Performance LC system (UPLC) equipped with binary solvent manager (Waters Corp., Milford, MA, USA), a UPLC BEH C18 column (1.7 μm, 2.1 mm × 50 mm, Waters Corp., Milford, MA, USA), and a Q-Tof Micro mass spectrometer (Waters, Manchester, UK) with electrospray ionization (ESI) source operating in negative and positive modes. The samples (10 μL) were injected, and the elution was completed in 15 min with a sequence of linear gradients and isocratic flow rates of 0.45 mL/min. The mobile phase consisted of solvent A (2.0% formic acid, v/v) and solvent B (100% acetonitrile). The program began with isocratic elution with 99% solvent A (0–1 min); then, a linear gradient was used until 12 min, lowering solvent A to 0%; from 12.5 to 13.5 min, the gradient returned to the initial composition (99% A), and then it was held constant to re-equilibrate the column. The data obtained from UPLC-MS were subsequently entered into the MassLynx 4.0 ChromaLynx Application Manager software. 

For the determination of phenolic compounds, a protocol described before by Lachowicz et al. [16,27] was followed. The runs were monitored at the following wavelengths: phenolic acids at 320 nm, flavonols at 360 nm, anthocyanins at 520 nm, and flavan-3-ols at 280 nm (Supplementary Materials S1). The photodiode array detector (PDA) spectra were measured over the wavelength range of 200–600 nm in steps of 2 nm. All data were obtained in triplicate. The results were expressed as mg/L chokeberry and cranberry fruit juices

### 3.9. Determination of Antioxidant Activity

The free-radical scavenging activities were determined using two methods, ABTS (radical scavenging capacity) and FRAP (ferric reducing antioxidative power). The ABTS and FRAP assays were conducted as previously described by Re et al. [39] and Benzie and Strain [40], respectively. Determinations by ABTS and FRAP methods were performed using a UV-2401 PC spectrophotometer (Shimadzu, Kyoto, Japan). The antioxidative activity was evaluated by measuring the variation in absorbance at 734 nm after 6 min for ABTS and at 593 nm after 10 min for FRAP. All antioxidative activity analyses were done in triplicate, and the results are expressed as mmol of Trolox equivalent (TE) per mL of sample.

### 3.10. Sensorian Evaluation

The sensory properties of the obtained fruit juices were evaluated using a nine-degree hedonic scale with boundary indications: “I do not like it extremely” (1) to “I like it extremely” (9). The assessment included the following quality attributes: taste, aroma, color, and turbidity. It was conducted by a group of 30 consumer panelists (15 men and 15 women in the age group of 20–65). Coded samples were provided to the panelists for the evaluation at 20 °C in uniform 50-mL plastic containers.

### 3.11. Statistical Analysis

Statistical analysis, including two-way ANOVA and the PCA, were conducted using Statistica 12.5 (StatSoft, Kraków, Poland). Significant differences (*p* ≤ 0.05) between means were assessed by Duncan’s t-test. 

## 4. Conclusions

To recapitulate, the additive in the form of CESP is an appropriate source of minerals, including Ca, for the enrichment of foods, including berry fruit juices. The juices from chokeberry and cranberry were enriched with macro- and microelements, and thus contained 25.7 and 66.3 times more Ca than the control samples. Juices supplementation with CESP had a significant effect on a decrease of their total acidity and their total organic acids content. The CESP supplementation at 1% caused no significant changes in the sediment content and color of both types of juices, but it significantly improved their taste. The addition of CESP to juices at increasing doses from 0.2% to 2% contributed to losses of polyphenolic compounds that ranged from 6.8% to 20.2% in the chokeberry juice and from 2.4% to 9,1% in the cranberry juice, respectively. Therefore, it would be advisable to decrease the CESP dose to 1% in order to minimize losses of polyphenols to 9.1% in the chokeberry juice and to 7.7% in the cranberry juice. On average, the losses of polyphenols determined for all additive doses reached 5.4% for the chokeberry juice and 3.4% for the cranberry juice. Pasteurization with the CESP additive did not impair juice enrichment with minerals and allowed preserving their biologically active polyphenolic compounds. In addition, the color of the heated juices turned lighter, whereas increasing doses of the additive made it become darker.

## Figures and Tables

**Table 1:** The change of CIE L*, a*, b* parameters in the products before and after thermal treatment. CESP: chicken eggshell powder.

CESP [%]	Chokeberry Juice						Cranberry Juice					
	Unheated			Heated			Unheated			Heated		
	L*	a*	b*	L*	a*	b*	L*	a*	b*	L*	a*	b*
0.0	49.09 ± 0.10^1^a^2^	60.80 ± 0.12a	37.65 ± 0.08a	49.52 ± 0.10a	59.58 ± 0.12a	35.70 ± 0.07c	78.50 ± 0.16h	33.68 ± 0.07a	12.31 ± 0.02a	76.74 ± 0.15h	35.21 ± 0.07a	13.01 ± 0.03a
0.2	49.06 ± 0.10b	58.81 ± 0.12b	29.69 ± 0.06b	37.26 ± 0.07d	58.88 ± 0.12b	43.32 ± 0.09a	80.27 ± 0.16g	29.96 ± 0.06b	10.60 ± 0.02b	79.62 ± 0.16g	29.64 ± 0.06b	10.36 ± 0.02b
0.4	48.15 ± 0.10d	56.56 ± 0.11i	23.31 ± 0.05d	39.53 ± 0.08c	57.41 ± 0.11d	30.31 ± 0.06e	81.56 ± 0.16f	26.81 ± 0.05c	9.26 ± 0.02c	82.27 ± 0.16f	24.10 ± 0.05c	8.39 ± 0.02c
0.6	48.58 ± 0.10c	55.03 ± 0.11k	20.28 ± 0.04i	29.79 ± 0.06i	56.76 ± 0.11h	37.68 ± 0.08b	82.75 ± 0.17e	24.07 ± 0.05d	8.26 ± 0.02d	83.06 ± 0.17e	21.46 ± 0.04d	7.45 ± 0.01d
0.8	41.79 ± 0.08h	58.63 ± 0.12c	24.85 ± 0.05c	40.53 ± 0.08b	58.44 ± 0.12c	25.05 ± 0.05j	81.81 ± 0.16e	21.62 ± 0.04e	7.93 ± 0.02e	84.41 ± 0.17d	18.33 ± 0.04e	6.56 ± 0.01e
1.0	39.05 ± 0.08j	57.87 ± 0.12h	21.22 ± 0.04f	35.78 ± 0.07e	55.33 ± 0.11j	23.07 ± 0.05k	84.41 ± 0.17c	20.40 ± 0.04f	6.86 ± 0.01f	85.28 ± 0.17c	15.93 ± 0.03f	5.96 ± 0.01f
1.2	41.71 ± 0.08h	58.35 ± 0.12d	23.58 ± 0.05e	32.45 ± 0.06f	57.14 ± 0.11e	27.70 ± 0.06g	84.00 ± 0.17c	18.61 ± 0.04g	6.61 ± 0.01g	85.66 ± 0.17c	15.01 ± 0.03g	5.58 ± 0.01h
1.4	44.02 ± 0.09e	57.05 ± 0.11g	21.05 ± 0.04h	29.01 ± 0.06j	56.98 ± 0.11g	33.87 ± 0.07d	84.90 ± 0.17c	18.00 ± 0.04h	6.16 ± 0.01h	84.29 ± 0.17d	13.98 ± 0.03h	5.61 ± 0.01g
1.6	40.07 ± 0.08i	58.02 ± 0.12e	21.71 ± 0.04g	27.90 ± 0.06k	55.98 ± 0.11i	29.40 ± 0.06f	83.29 ± 0.17d	13.29 ± 0.03j	5.90 ± 0.01i	87.20 ± 0.17a	11.07 ± 0.02i	4.89 ± 0.01j
1.8	42.62 ± 0.09f	55.99 ± 0.11j	17.50 ± 0.04k	30.74 ± 0.06h	57.13 ± 0.11e	27.19 ± 0.05h	85.90 ± 0.17b	15.17 ± 0.03i	5.00 ± 0.01j	86.91 ± 0.17b	10.99 ± 0.02j	4.81 ± 0.01k
2.0	42.34 ± 0.08g	56.67 ± 0.11h	19.16 ± 0.04j	31.29 ± 0.06g	57.03 ± 0.11f	27.05 ± 0.05i	86.50 ± 0.17a	13.77 ± 0.03j	4.75 ± 0.01k	84.42 ± 0.17d	14.64 ± 0.03g	5.09 ± 0.01i

^1^ Values are means ± standard deviation. *n* = 3; ^2^ different letters represent significant differences (*p* < 0.05).

**Table 2:** The change of organic acid in the products before and after thermal treatment.

Process	CESP [%]	Chokeberry Juice						Cranberry Juice					
		OA	CA	ICA	MA	QA	FA	OA	GA	CA	ICA	MA	SA
Unheated	0.0	91.3 ± 0.0d	322.3 ± 0.1m	968.2 ± 0.2c	15482.6 ± 3.1h	3224.0 ± 0.6c	677.9 ± 0.1f	358.6 ± 0.1f	161.3 ± 0.0e	24364.4 ± 4.9h	1666.4 ± 0.3h	17435.9 ± 3.5h	6162.2 ± 1.2f
	0.2	90.0 ± 0.0d	236.0 ± 0.0l	1022.8 ± 0.2b	15256.7 ± 3.1i	3221.8 ± 0.6c	662.9 ± 0.1g	359.0 ± 0.1f	145.8 ± 0.0h	24152.9 ± 4.8i	1661.8 ± 0.3h	17401.6 ± 3.5i	6145.5 ± 1.2f
	0.4	96.3 ± 0.0c	268.6 ± 0.1p	568.1 ± 0.1i	16367.9 ± 3.3d	3023.8 ± 0.6d	736.4 ± 0.1d	353.5 ± 0.1g	160.6 ± 0.0e	23755.8 ± 4.8j	1648.6 ± 0.3i	17137.4 ± 3.4j	6026.9 ± 1.2g
	0.6	85.8 ± 0.0e	250.2 ± 0.1s	486.6 ± 0.1o	14462.8 ± 2.9l	2938.9 ± 0.6e	652.6 ± 0.1h	344.8 ± 0.1h	167.1 ± 0.0d	23318.7 ± 4.7l	1617.5 ± 0.3l	16799.2 ± 3.4l	5896.6 ± 1.2h
	0.8	81.8 ± 0.0g	387.0 ± 0.1i	635.2 ± 0.1e	14533.6 ± 2.9k	2490.6 ± 0.5i	635.2 ± 0.1i	334.3 ± 0.1i	145.3 ± 0.0h	22677.7 ± 4.5m	1573.5 ± 0.3n	16334.9 ± 3.3o	5729.6 ± 1.1i
	1.0	76.0 ± 0.0i	365.2 ± 0.1k	477.8 ± 0.1	14364.9 ± 2.9m	2454.0 ± 0.5i	575.1 ± 0.1n	337.9 ± 0.1i	147.0 ± 0.0h	22827.8 ± 4.6m	1591.0 ± 0.3m	16415.5 ± 3.3n	5765.5 ± 1.2i
	1.2	80.0 ± 0.0g	381.1 ± 0.1j	545.7 ± 0.1k	13914.4 ± 2.8p	2435.3 ± 0.5i	602.9 ± 0.1k	343.5 ± 0.1h	154.3 ± 0.0g	23244.9 ± 4.6l	1632.4 ± 0.3j	16660.7 ± 3.3m	5858.0 ± 1.2h
	1.4	75.7 ± 0.0i	375.2 ± 0.1j	543.5 ± 0.1k	13269.0 ± 2.7r	2267.0 ± 0.5k	565.2 ± 0.1o	345.1 ± 0.1h	149.3 ± 0.0h	23343.7 ± 4.7l	1637.2 ± 0.3j	16793.3 ± 3.4l	5894.1 ± 1.2h
	1.6	79.7 ± 0.0h	477.4 ± 0.1e	507.3 ± 0.1n	14187.5 ± 2.8n	2390.9 ± 0.5j	577.9 ± 0.1n	361.5 ± 0.1e	176.5 ± 0.0c	23650.2 ± 4.7k	1620.7 ± 0.3k	17152.3 ± 3.4j	5890.2 ± 1.2h
	1.8	73.9 ± 0.0j	525.9 ± 0.1d	558.8 ± 0.1j	14979.5 ± 3.0j	2384.9 ± 0.5j	541.3 ± 0.1p	352.7 ± 0.1g	159.0 ± 0.0f	23984.8 ± 4.8j	1679.8 ± 0.3g	17032.1 ± 3.4k	6001.5 ± 1.2g
	2.0	79.4 ± 0.0h	557.8 ± 0.1c	601.4 ± 0.1g	14022.4 ± 2.8o	2349.1 ± 0.5j	580.6 ± 0.1m	369.7 ± 0.1g	169.2 ± 0.0f	24484.1 ± 4.8j	1790.2 ± 0.3g	18012.2 ± 3.4k	6211.2 ± 1.2g
Heated	0.0	100.5 ± 0.0b	354.6 ± 0.1r	1065.0 ± 0.2a	17030.9 ± 3.4b	3546.4 ± 0.7a	745.7 ± 0.1b	394.5 ± 0.1a	177.4 ± 0.0c	26800.8 ± 5.4a	1833.1 ± 0.4b	19179.5 ± 3.8a	6778.4 ± 1.4a
	0.2	99.0 ± 0.0b	259.7 ± 0.1r	1125.0 ± 0.2b	16782.4 ± 3.4c	3543.9 ± 0.7a	729.2 ± 0.1c	394.9 ± 0.1a	160.3 ± 0.0e	26568.1 ± 5.3b	1828.0 ± 0.4b	19141.7 ± 3.8a	6760.0 ± 1.4a
	0.4	105.9 ± 0.0a	295.5 ± 0.1o	624.9 ± 0.1e	18004.7 ± 3.6a	3326.2 ± 0.7b	810.0 ± 0.2a	388.8 ± 0.1b	176.6 ± 0.0c	26131.3 ± 5.2d	1813.5 ± 0.4c	18851.2 ± 3.8b	6629.6 ± 1.3b
	0.6	94.4 ± 0.0c	275.2 ± 0.1p	535.3 ± 0.1l	15909.1 ± 3.2e	3232.8 ± 0.6b	717.9 ± 0.1d	379.3 ± 0.1c	183.8 ± 0.0b	25650.6 ± 5.1e	1779.2 ± 0.4e	18479.1 ± 3.7d	6486.3 ± 1.3c
	0.8	90.0 ± 0.0d	425.7 ± 0.1f	698.8 ± 0.1d	15987.0 ± 3.2e	2739.6 ± 0.5f	698.7 ± 0.1e	367.7 ± 0.1e	159.9 ± 0.0f	24945.4 ± 5.0g	1730.9 ± 0.3f	17968.4 ± 3.6g	6302.6 ± 1.3e
	1.0	83.6 ± 0.0f	401.7 ± 0.1h	525.6 ± 0.1m	15801.4 ± 3.2f	2699.4 ± 0.5g	632.6 ± 0.1i	371.7 ± 0.1d	161.7 ± 0.0e	25110.6 ± 5.0f	1750.1 ± 0.4f	18057.1 ± 3.6f	6342.1 ± 1.3e
	1.2	88.0 ± 0.0e	419.2 ± 0.1f	600.3 ± 0.1g	15305.8 ± 3.1i	2678.8 ± 0.5g	663.2 ± 0.1g	377.9 ± 0.1c	169.7 ± 0.0d	25569.4 ± 5.1e	1795.6 ± 0.4e	18326.8 ± 3.7e	6443.8 ± 1.3d
	1.4	83.2 ± 0.0f	412.7 ± 0.1g	597.8 ± 0.1h	14596.0 ± 2.9k	2493.7 ± 0.5i	621.7 ± 0.1j	379.7 ± 0.1c	164.2 ± 0.0e	25678.1 ± 5.1e	1800.9 ± 0.4d	18472.6 ± 3.7d	6483.5 ± 1.3c
	1.6	87.7 ± 0.0e	525.1 ± 0.1d	558.1 ± 0.1j	15606.3 ± 3.1g	2629.9 ± 0.5g	635.7 ± 0.1i	397.6 ± 0.1a	194.2 ± 0.0a	26015.2 ± 5.2d	1782.8 ± 0.4e	18867.6 ± 3.8b	6479.2 ± 1.3c
	1.8	81.3 ± 0.0g	578.5 ± 0.1b	614.7 ± 0.1f	16477.5 ± 3.3d	2623.4 ± 0.5g	595.4 ± 0.1l	388.0 ± 0.1b	174.9 ± 0.0c	26383.3 ± 5.3b	1847.7 ± 0.4a	18735.4 ± 3.7c	6601.6 ± 1.3b
	2.0	87.3 ± 0.0e	613.6 ± 0.1a	661.6 ± 0.1e	15424.6 ± 3.1h	2584.0 ± 0.5h	638.6 ± 0.1i	398.0 ± 0.1b	181.2 ± 0.0c	27213.2 ± 5.3b	1900.7 ± 0.4a	19105.4 ± 3.7c	6701.3 ± 1.3b

^1^ Values are means ± standard deviation. *n* = 3; *Explanation:* OA, oxalic acid; CA, citric acid; ICA, isocitric acid; MA, malic acid; QA, quinic acid; FA, ferulic acid; GA, galacturonic acid; SA, succinic acid.

**Table 3:** Minerals content in CESP.

Minerals	Sum [mg/100 g]	Ratio of Minerals [%]
Al	0.00	0.00
As	0.03	0.00
Ca	27695.83	27.70
Cd	0.01	0.00
Cr	0.00	0.00
Cu	0.08	0.00
Fe	0.52	0.00
K	57.59	0.06
Mg	352.28	0.35
Mn	0.12	0.00
Mo	0.00	0.00
Na	103.89	0.10
Ni	0.00	0.00
P	85.53	0.09
Pb	0.00	0.00
S	94.78	0.10
Sr	22.35	0.02
Zn	0.00	0.00

**Table 4:** Identification of polyphenolic compounds.

Tentative Identification	λmax [nm]	[H − M]− (*m*/*z*)	MS/MS Fragments (*m*/*z*)	Chokeberry	Cranberry
Cyanidin-3-hexoside-(epi)catechin	520	737+	575/423/287	x	
Neochlorogenic acid	323	353	191	x	
Cyanidin-3-pentoside-(epi)catechin	520	707+	557/329/287	x	
( + )-catechin	280	289	245/203		x
Cyanidin-3-hexoside-(epi)cat-(epi)cat	520	1025+	575/409/287	x	
3-*O*-*p*-Coumaroylquinic acid	310	337	191	x	x
Delfinidyn-3-*O*-glucoside	520	465+	303		x
*p*-Coumaroyl-hexose isomer	310	325	163		x
*p*-Coumaroyl-hexose isomer	310	325	163		x
B-type procyanidin-dimer	280	577	289		x
Cyanidin-3-*O*-galactoside	516	449+	287	x	x
Chlorogenic acid	323	353	191	x	x
Cryptochlorogenic acid	323	353	191	x	
Caffeoyl dihexoside	320	503	341/179		x
Caffeoyl hexoside	320	341	179		x
Caffeoyl hexoside isomer	320	341	179		x
Cyanidin-3-*O*-glucoside	517	449+	287	x	x
Sinapoyl-hexose	320	385	223		x
Delphinidin-3-*O*-arabinoside	520	435	303		x
Peonidin-3-*O*-galactoside	515	463+	301		x
Cyanidin-3-*O*-arabinoside	515	419+	287+	x	x
(−)-Epicatechin	280	289	245/203		x
A-type PA-trimer	280	863	289		x
Cyanidin-3-*O*-xyloside	515	419+	287+	x	
Peonidin-3-*O*-glucoside	515	463+	301		x
Peonidin-3-*O*-arabinoside	515	433+	301		x
Malvidin-3-O-galactoside	515	493	331		x
Malwidin-3-*O*-arabinoside	520	463+	331		x
A-type PA-tetramer	280	1151	289		x
Quercetin-dihexoside	352	625	445/301	x	
Quercetin-dihexoside	352	625	445/301	x	
Quercetin-3-*O*-vicianoside	353	595	432/301	x	
Quercetin-3-*O-*robinobioside	353	609	463/301	x	
Quercetin-3-*O*-rutinoside	353	609	463/301	x	x
Myricetin-3-O-arabinoside	355	449	317		x
Myricetin-3-O-galactoside	355	479	317		x
Quercetin-3-*O*-galactoside	352	463	301	x	x
Quercetin-3-*O*-glucoside	352	463	301	x	x
Myricetin-3-O-glucoside	355	479	317		x
Eriodictyol-glucuronide	280	463	287	x	
Isorhamnetin pentosylhexoside	352	609	315	x	
Quercetin-*O*-deoxyhexose-deoxyhexoside	352	593	433/301	x	
Isorhamnetin rhamnosyl hexoside isomer	352	623	463/315	x	
Isorhamnetin rhamnosyl hexoside isomer	352	623	421/315	x	
Isorhamnetin-3-*O*-galactoside	350	477	315		x
Syringetin-3-*O*-galactoside	358	507	330		x
Methoxyquercetin-pentoside	350	447	315		x
Isorhamnetin-3-*O*-arabinoside	352	447	315		x
Quercetin-3-*O*-(6”*p*-coumaroyl)-galactoside	350	609	301		x

**Table 5:** The antioxidant activity of products. ABTS: 2,2′-azinobis(3-ethylbenzothiazoline-6-sulfonic acid), FRAP: ferric reducing antioxidative power.

Addition [%]	Antioxidant Activity [mmol TE/mL]							
	FRAP				ABTS			
	Unheated		Heated		Unheated		Heated	
	Cranberry	Chokeberry	Cranberry	Chokeberry	Cranberry	Chokeberry	Cranberry	Chokeberry
0.0	1.38 ± 0.00^1^a^2^	10.94 ± 0.02A	1.31 ± 0.00b	9.98 ± 0.02B	1.48 ± 0.00a	9.79 ± 0.02A	1.41 ± 0.00b	9.16 ± 0.02B
0.2	1.01 ± 0.00g	7.61 ± 0.02B	1.00 ± 0.00g	7.47 ± 0.01C	1.16 ± 0.00d	7.32 ± 0.01C	1.03 ± 0.00g	7.27 ± 0.02C
0.4	1.19 ± 0.00c	6.88 ± 0.01G	1.18 ± 0.00c	6.82 ± 0.01G	1.25 ± 0.00c	7.08 ± 0.01D	1.21 ± 0.00c	7.01 ± 0.01D
0.6	1.03 ± 0.00g	5.69 ± 0.01K	1.00 ± 0.00c	5.38 ± 0.01L	1.15 ± 0.00d	5.27 ± 0.01I	1.05 ± 0.00g	5.20 ± 0.01I
0.8	1.18 ± 0.00c	5.14 ± 0.01M	1.11 ± 0.00e	5.11 ± 0.01M	1.09 ± 0.00f	5.07 ± 0.01J	1.01 ± 0.00gh	5.05 ± 0.01J
1.0	1.06 ± 0.00f	6.45 ± 0.01I	1.03 ± 0.00g	6.05 ± 0.01J	1.14 ± 0.00d	4.95 ± 0.01J	1.08 ± 0.00f	4.58 ± 0.01K
1.2	1.32 ± 0.00b	6.10 ± 0.01J	1.11 ± 0.00e	5.64 ± 0.01K	1.09 ± 0.00f	5.53 ± 0.01H	1.05 ± 0.00g	5.22 ± 0.01I
1.4	0.88 ± 0.00j	7.29 ± 0.01D	0.84 ± 0.00k	7.20 ± 0.01D	1.02 ± 0.00g	7.06 ± 0.01D	0.90 ± 0.00i	7.03 ± 0.01D
1.6	1.05 ± 0.00f	7.04 ± 0.01F	1.01 ± 0.00g	6.55 ± 0.01H	0.99 ± 0.00h	6.42 ± 0.01F	0.97 ± 0.00h	6.38 ± 0.01F
1.8	1.18 ± 0.00c	7.44 ± 0.01C	1.15 ± 0.00d	7.21 ± 0.01D	1.13 ± 0.00e	7.07 ± 0.01D	1.11 ± 0.00e	7.03 ± 0.02D
2.0	0.94 ± 0.00h	7.11 ± 0.01E	0.91 ± 0.00i	6.63 ± 0.01H	1.09 ± 0.00f	6.50 ± 0.01E	0.96 ± 0.00h	6.25 ± 0.01G

^1^ Values are means ± standard deviation. *n* = 3; ^2^ a-j and A-M - different letters represent significant differences between doses of CESP and thermal treatment (*p* < 0.05).

## Supplementary Materials

The following are available online, Table S1: The content of polyphenolic compounds in the chokeberry juice with CESP [mg/100 mL]; Table S2: The content of polyphenolic compounds in the cranberry juice with CESP [mg/100 mL].
