# Supplementary material for: Impact Mineralization of Chokeberry and Cranberry Fruit Juices Using a New Functional Additive on the Protection of Bioactive Compounds and Antioxidative Properties
Source: Molecules. 2020 Feb 4;25(3):659. doi: 10.3390/molecules25030659 (PMC7037092; doi:10.3390/molecules25030659)
Supplement: Supplementary file 1 [file molecules-25-00659-s001.pdf]

molecules

MDPI

Type of the Paper (<u>Article</u>, Review, Communication, etc.)

Impact mineralization of chokeberry and cranberry fruit juices using a new functional additive on the protection bioactive compounds and antioxidative properties

Sabina Lachowicz [1,*], Jan Oszmiański [2], Martyna Wilczyńska [2], Grzegorz Zaguła [3], Bogdan Saletnik [3] and Czesław Puchalski [3]

[1] Department of Fermentation and Cereals Technology, Faculty of Biotechnology and Food Science, Wrocław University of Environmental and Life Science, 37, Chełmońskiego Street, 51-630 Wroclaw, Poland, Sabina.lachowicz@upwr.edu.pl,

[2] Department of Fruit, Vegetable and Plant Nutraceutical Technology, Faculty of Biotechnology and Food Science, Wrocław University of Environmental and Life Sciences, 37, Chełmońskiego Street, 51-630 Wrocław, Poland; jan.oszmianski@upwr.edu.pl (J.O.); martyna28b@gmail.com (M.W.),

[3] Department of Bioenergetics and Food Analysis, Faculty of Biology and Agriculture, University of Rzeszów, 35-601 Rzeszów, Poland; g_zagula@univ.rzeszow.pl (G.Z.); cpuchal@ur.edu.pl (Cz.P.); bogdan.saletnik@ur.edu.pl (B.S).

* Correspondence: Sabina Lachowicz, sabina.lachowicz@upwr.edu.pl

Received: 9 January 2020; Accepted: date; Published: date

Table S1. The content of polyphenolic compounds in the chokeberry juice with CESP [mg/100 mL]

Com.	Unheted											Heated										
	0.0%	0.2%	0.4%	0.6%	0.8%	1.0%	1.2%	1.4%	1.6%	1.8%	2.0%	0.0%	0.2%	0.4%	0.6%	0.8%	1.0%	1.2%	1.4%	1.6%	1.8%	2.0%
C-3-he	25.8±0.1	29.0±0.1	27.2±0.1	25.7±0.1	26.0±0.1	26.5±0.1	26.9±0.1	24±0.0	24.6±0.0	22.7±0.0	19.7±0.0	22.7±0.0	23.6±0.0	26.3±0.1	24.4±0.0	24.6±0.0	21.2±0.0	22.3±0.0	22.6±0.0	22.3±0.0	20.6±0.0	23.0±0.0
C-3-pe	16.9±0.0	19.7±0.0	21.6±0.0	22.1±0.0	19.1±0.0	23.0±0.0	16.6±0.0	22.4±0.0	21.6±0.0	22.3±0.0	18.2±0.0	12.3±0.0	13.1±0.0	12.9±0.0	11.6±0.0	10.9±0.0	10.6±0.0	12.8±0.0	12.9±0.0	12.5±0.0	12.8±0.0	14.0±0.0
C-3-hee	14.2±0.0	14.2±0.0	13.9±0.0	11.8±0.0	11.5±0.0	8.6±0.0	10.1±0.0	11.9±0.0	10.8±0.0	12.8±0.0	9.2±0.0	12.2±0.0	12.4±0.0	13.7±0.0	12.5±0.0	8.4±0.0	9.9±0.0	11.6±0.0	10.9±0.0	11.4±0.0	9.1±0.0	10.1±0.0
C-3-gal	2600.5±5.2	2820.2±5.6	2549.6±5.1	2514.1±5.0	2505.1±5.0	2508.1±5.0	2515.0±5.0	2468.5±4.9	2450±4.9	2330.2±4.7	2006.5±4.0	2535.3±5.1	2575.9±5.2	2743.2±5.5	2546.2±5.1	2579.5±5.2	2324±4.6	2417.4±4.8	2319.4±4.6	2350.7±4.7	2212.3±4.4	2366.7±4.7
C-3-glu	109.4±0.2	119±0.2	106.2±0.2	105.3±0.2	105.8±0.2	107.4±0.2	106.3±0.2	102.1±0.2	102.9±0.2	95.9±0.2	84.8±0.2	106.2±0.2	107.5±0.2	114.6±0.2	106.3±0.2	106.5±0.2	96.4±0.2	99.5±0.2	95.8±0.2	97.3±0.2	93±0.2	99.2±0.2
C-3-ara	911.5±1.8	996.4±2.0	889.7±1.8	877.9±1.8	876.0±1.8	876.3±1.8	889.4±1.8	861.2±1.7	854.8±1.7	811.5±1.6	695.6±1.4	903.3±1.8	913.7±1.8	973.2±1.9	905.9±1.8	916.7±1.8	823.5±1.6	856.5±1.7	819.9±1.6	831.7±1.7	775.6±1.6	837.5±1.7
C-3-xyl	10.4±0.0	10.8±0.0	10.1±0.0	9.4±0.0	10.7±0.0	10.0±0.0	8.8±0.0	9.2±0.0	8.8±0.0	9.2±0.0	8.4±0.0	9.2±0.0	11±0.0	11.2±0.0	10.2±0.0	11.3±0.0	8.4±0.0	10.8±0.0	9.2±0.0	8.6±0.0	8.7±0.0	8.4±0.0
C	113±0.2	123.4±0.2	112.3±0.2	109.1±0.2	110.6±0.2	113.2±0.2	114.6±0.2	105.5±0.2	108.4±0.2	101±0.2	85.4±0.2	110.2±0.2	114.6±0.2	119.7±0.2	113.2±0.2	114.9±0.2	101.5±0.2	105.6±0.2	103.5±0.2	103.1±0.2	103.5±0.2	107.9±0.2
3CQ	1429.5±2.9	1514.4±3	1431.2±2.9	1384.4±2.8	1379.2±2.8	1379.3±2.8	1358±2.7	1373.9±2.7	1348.9±2.7	1291±2.6	1106.7±2.2	1292.3±2.6	1318.7±2.6	1407.2±2.8	1309.1±2.6	1338.1±2.7	1218.7±2.4	1256.4±2.5	1202.7±2.4	1218.3±2.4	1155.5±2.3	1230.8±2.5
pCQ	36±0.1	38±0.1	42.4±0.1	42±0.1	41.2±0.1	41.8±0.1	40.5±0.1	40.3±0.1	40.2±0.1	38.1±0.1	32.3±0.1	32.6±0.1	33±0.1	44.4±0.1	32.7±0.1	37.8±0.1	30.9±0.1	31.6±0.1	30.2±0.1	30.7±0.1	29.2±0.1	30.8±0.1
5CQ	1439±2.9	1525.2±3.1	1449.3±2.9	1404.5±2.8	1408.1±2.8	1411.7±2.8	1375.7±2.8	1389.6±2.8	1373.8±2.7	1311.4±2.6	1130.1±2.3	1301.2±2.6	1326.6±2.7	1415.3±2.8	1314.9±2.6	1541.8±3.1	1224±2.4	1260.8±2.5	1207±2.4	1223.7±2.4	1155.4±2.3	1233.3±2.5
4CQ	76.3±0.2	78.7±0.2	85.2±0.2	92±0.2	101.9±0.2	103.1±0.2	78.2±0.2	85.3±0.2	96.9±0.2	85.4±0.2	86.4±0.2	65.6±0.1	66.9±0.1	71.6±0.1	65.6±0.1	66.7±0.1	60.5±0.1	62.8±0.1	60.0±0.1	61.2±0.1	58.6±0.1	61.7±0.1
Qdih	33.2±0.1	35.8±0.1	32.2±0.1	32.4±0.1	32.8±0.1	32.1±0.1	33.7±0.1	30.8±0.1	26.1±0.1	32.2±0.1	32.2±0.1	29.4±0.1	30.4±0.1	31.6±0.1	29.9±0.1	19.0±0.0	28.0±0.1	29.5±0.1	28.3±0.1	28.6±0.1	26.0±0.1	28.0±0.1
Qdih	12.0±0.0	12.6±0.0	11.2±0.0	12±0.0	11.5±0.0	11.7±0.0	12.2±0.0	10.9±0.0	9.8±0.0	11.7±0.0	11.7±0.0	10±0.0	10.4±0.0	11.1±0.0	10.7±0.0	6.5±0.0	10.2±0.0	10.5±0.0	10.1±0.0	10.1±0.0	9.4±0.0	10.2±0.0
Q-3-vic	66.4±0.1	70.1±0.1	63.7±0.1	63.2±0.1	65.7±0.1	64.7±0.1	67.5±0.1	60.1±0.1	52±0.1	65.4±0.1	65.4±0.1	59.1±0.1	60.6±0.1	64.6±0.1	60.5±0.1	39.2±0.1	56±0.1	58.5±0.1	56±0.1	56.7±0.1	53.1±0.1	56.9±0.1
Q-3-rob	60.5±0.1	65±0.1	59.2±0.1	58.5±0.1	60.7±0.1	58.8±0.1	60.6±0.1	55.8±0.1	47.5±0.1	60.7±0.1	60.5±0.1	55.7±0.1	57.5±0.1	62.1±0.1	56.8±0.1	36.8±0.1	52.8±0.1	55.7±0.1	52.3±0.1	53.1±0.1	50.4±0.1	54.3±0.1
Q-3-rut	62.0±0.1	64.4±0.1	62.5±0.1	60.1±0.1	60.7±0.1	59.5±0.1	63.7±0.1	59.2±0.1	49.7±0.1	62.8±0.1	62.2±0.1	52.9±0.1	54.8±0.1	58.4±0.1	54.7±0.1	34.6±0.1	51.8±0.1	53.2±0.1	50.9±0.1	52.1±0.1	49±0.1	52.2±0.1
Q-3-gal	145.4±0.3	153.5±0.3	139.4±0.3	137.5±0.3	139.4±0.3	138.8±0.3	143.8±0.3	131.5±0.3	112.2±0.2	140.9±0.3	139.9±0.3	131.1±0.3	133.2±0.3	141.5±0.3	131±0.3	85.2±0.2	123±0.2	126.5±0.3	121.9±0.2	122.2±0.2	116.3±0.2	123.9±0.2
Q-3-3glu	95.3±0.2	102.7±0.2	92.3±0.2	93.1±0.2	92.6±0.2	91.6±0.2	94.7±0.2	87.1±0.2	74.8±0.1	94.4±0.2	92±0.2	87.7±0.2	86.6±0.2	93.6±0.2	86.1±0.2	57±0.1	84.5±0.2	84±0.2	83.8±0.2	81.4±0.2	76.6±0.2	83±0.2
Ipenh	4.3±0.0	4.2±0.0	4.9±0.0	4.7±0.0	4.7±0.0	4.3±0.0	4.4±0.0	4.4±0.0	3.9±0.0	4.3±0.0	4.0±0.0	4.1±0.0	4.6±0.0	5.2±0.0	4.3±0.0	3.6±0.0	3.9±0.0	3.6±0.0	3.8±0.0	4.8±0.0	3.6±0.0	3.3±0.0
Qdideoxyh	9.0±0.0	9.4±0.0	8.3±0.0	9.1±0.0	9.3±0.0	8.6±0.0	10.2±0.0	8.6±0.0	7.1±0.0	8.6±0.0	8.5±0.0	9.5±0.0	8.6±0.0	8.1±0.0	7.6±0.0	5.9±0.0	7.2±0.0	8.1±0.0	7.6±0.0	7.2±0.0	7.4±0.0	7.6±0.0

Irhahex	4.8±0.0	6.2±0.0	4.5±0.0	4.8±0.0	4.8±0.0	5.3±0.0	5.7±0.0	4.7±0.0	3.6±0.0	4.6±0.0	4.8±0.0	5.3±0.0	4.7±0.0	5.0±0.0	5.1±0.0	1.8±0.0	5.1±0.0	5.1±0.0	4.5±0.0	3.8±0.0	3.9±0.0	4.5±0.0
Irhahex	3.5±0.0	3.8±0.0	3.8±0.0	3.6±0.0	3.5±0.0	3.3±0.0	3.0±0.0	3.1±0.0	3.0±0.0	3.2±0.0	3.3±0.0	3.2±0.0	2.8±0.0	3.6±0.0	2.5±0.0	2.0±0.0	3.1±0.0	3.0±0.0	3.4±0.0	2.9±0.0	3.1±0.0	2.8±0.0
Egluc	8.2±0.0	7.6±0.0	6.2±0.0	6.2±0.0	5.1±0.0	5.5±0.0	5.4±0.0	5.0±0.0	4.4±0.0	5.1±0.0	5.9±0.0	4.7±0.0	5.0±0.0	5.5±0.0	4.4±0.0	2.9±0.0	4.8±0.0	5.2±0.0	4.8±0.0	4.5±0.0	4.2±0.0	5.5±0.0
**SUM PC**	7286.9± 14.6	7824.2± 15.6	7255±1 4.5	7099.5± 14.2	7100±1 4.2	7106.8± 14.2	7055.7± 14.1	6980.7 ±14	6855.4± 13.7	6647.9± 13.3	5789.2± 11.6	6855.6± 13.7	6976.1 ±14	7443.6± 14.9	6906.2± 13.8	7051.4± 14.1	6360.1± 12.7	6590.9± 13.2	6321.6± 12.6	6398.9± 12.8	6037.3± 12.1	6455.4± 12.9

[1] Values are means ± standard deviation. n = 3;

Explanations:*- C-3-he, Cyanidin-3-hexoside-(epi)catechin; C-3-pe, Cyanidin-3-pentoside-(epi)catechin(+)-catechin; C-3-hec, Cyanidin-3-hexoside-(epi)cat-(epi)cat; C-3-gal, Cyanidin-3-*O*-galactoside; C-3-glu, Cyanidin-3-*O*-glucoside; C-3-ara, Cyanidin-3-*O*-arabinoside; C-3-xyl, Cyanidin-3-*O*-xyloside; C, Cyanidin; 3CQ, Neochlorogenic acid; pCQ, *p*-Coumaroyl-hexose isomer; 5CQ, Chlorogenic acid; 4CQ, Cryptochlorogenic acid; Qdih, Quercetin-dihexoside; Q-3-vic, Quercetin-3-*O*-vicianoside; Q-3-rob, Quercetin-3-*O*-robinobioside; Q-3-rut, Quercetin-3-*O*-rutinoside; Q-3-gal, Quercetin-3-*O*-galactoside; Q-3-glu, Quercetin-3-*O*-glucoside; Ipenh, Isorhamnetin pentosylhexoside; Qdideoxyh, Quercetin-*O*-deoxyhexose-deoxyhexoside; Irhahex, Isorhamnetin rhamnosyl hexoside isomer; Egluc, Eriodictyol-glucuronide.

Table S2. The content of polyphenolic compounds in the cranberry juice with CESP [mg/100 mL]

Com.	Unhetaed											Heated										
	0.0%	0.2%	0.4%	0.6%	0.8%	1.0%	1.2%	1.4%	1.6%	1.8%	2.0%	0.0%	0.2%	0.4%	0.6%	0.8%	1.0%	1.2%	1.4%	1.6%	1.8%	2.0%
D-3-gal	27.9±0.1	38.7±0.1	37.5±0.1	37.6±0.1	36.9±0.1	35.1±0.1	36±0.1	36.1±0.1	34.7±0.1	34.3±0.1	33.8±0.1	35±0.1	34.8±0.1	33.5±0.1	33±0.1	31.4±0.1	32.6±0.1	31.2±0.1	32.2±0.1	30.8±0.1	29.4±0.1	29.1±0.1
C-3-gal	4.1±0.0	6.5±0.0	5.9±0.0	6.3±0.0	6.3±0.0	6.1±0.0	6.0±0.0	6.2±0.0	6.3±0.0	6.1±0.0	5.7±0.0	6.2±0.0	6.0±0.0	5.8±0.0	5.8±0.0	5.3±0.0	5.2±0.0	7.7±0.0	5.6±0.0	5.5±0.0	5.0±0.0	5.0±0.0
C-3-glu	5.8±0.0	9.2±0.0	8.8±0.0	9.0±0.0	8.8±0.0	8.7±0.0	8.5±0.0	8.9±0.0	9.0±0.0	8.1±0.0	8±0.0	8.4±0.0	8.7±0.0	8.4±0.0	8.4±0.0	7.5±0.0	7.5±0.0	31.5±0.1	7.5±0.0	7.4±0.0	7.1±0.0	6.4±0.0
D-3-ara	28.2±0.1	41±0.1	38.7±0.1	38.4±0.1	38±0.1	36.8±0.1	37.9±0.1	37.8±0.1	36.3±0.1	35.3±0.1	35.8±0.1	35.7±0.1	34.9±0.1	33.6±0.1	33.2±0.1	31.9±0.1	33.2±0.1	82.9±0.2	32±0.1	30.2±0.1	29.7±0.1	28.6±0.1
P-3-gal	71.6±0.1	103.3±0.2	100.6±0.2	100.2±0.2	98.1±0.2	95.6±0.2	94.3±0.2	97.3±0.2	92.5±0.2	90.7±0.2	89.9±0.2	93.1±0.2	91.7±0.2	89.2±0.2	87.6±0.2	83.2±0.2	85.6±0.2	7.6±0.0	83.5±0.2	80.6±0.2	77.3±0.2	75.6±0.2
C-3-ara	6.9±0.0	9.7±0.0	9.8±0.0	9.4±0.0	9.3±0.0	8.8±0.0	9.1±0.0	9.3±0.0	9.6±0.0	9±0.0	8.3±0.0	8.3±0.0	8.7±0.0	8.2±0.0	8.7±0.0	7.2±0.0	8.3±0.0	51.5±0.1	7.8±0.0	8.0±0.0	7.8±0.0	7.5±0.0
P-3-glu	44.4±0.1	65.8±0.1	63.8±0.1	63.5±0.1	61.7±0.1	60.2±0.1	60.4±0.1	61.4±0.1	58.5±0.1	57.5±0.1	57.3±0.1	57.4±0.1	56.7±0.1	54.2±0.1	53.7±0.1	51.2±0.1	52.3±0.1	1.7±0.0	51±0.1	49.5±0.1	47.4±0.1	46.7±0.1
M-3-gal	1.8±0.0	2.6±0.0	2±0.0	2.3±0.0	2.2±0.0	3.1±0.0	1.9±0.0	2.5±0.0	1.7±0.0	2±0.0	1.9±0.0	2.2±0.0	2±0.0	2.1±0.0	2.3±0.0	1.9±0.0	2.0±0.0	0.5±0.0	1.9±0.0	2.1±0.0	1.4±0.0	1.8±0.0
Myr-3-ara	13±0.0	18.6±0.0	17.7±0.0	18.2±0.0	18.1±0.0	17.7±0.0	17.7±0.0	18.5±0.0	17.3±0.0	17.6±0.0	17.3±0.0	18.2±0.0	19.7±0.0	18.1±0.0	18.8±0.0	17.8±0.0	18.9±0.0	18.4±0.0	18.8±0.0	17.8±0	17.8±0.0	17.2±0.0
Myr-3-gal	3.1±0.0	4.7±0.0	4.9±0.0	5.0±0.0	5.1±0.0	5.0±0.0	5.2±0.0	5.6±0.0	4.9±0.0	5.5±0.0	5.3±0.0	5.3±0.0	5.4±0.0	5.1±0.0	5.1±0.0	5.1±0.0	5.4±0.0	5.5±0.0	5.5±0.0	5.6±0.0	5.1±0.0	5.2±0.0
Myr-3-glu	7.1±0.0	7.9±0.0	8.3±0.0	8.9±0.0	9.7±0.0	7.3±0.0	7.6±0.0	8.1±0.0	8.8±0.0	8.6±0.0	8.2±0.0	9.6±0.0	8.5±0.0	8.4±0.0	8.6±0.0	8.1±0.0	7.8±0.0	9.1±0.0	9.1±0.0	8.6±0.0	8.6±0.0	7.9±0.0
Q-3-gal	2.3±0.0	2.8±0.0	2.1±0.0	2.8±0.0	3.0±0.0	2.0±0.0	2.0±0.0	2.1±0.0	3.0±0.0	2.2±0.0	2.3±0.0	3.6±0.0	2.7±0.0	2.6±0.0	3.1±0.0	2.5±0.0	2.5±0.0	2.6±0.0	2.6±0.0	2.8±0.0	2.7±0.0	2.4±0.0
Q-3-glu	14.2±0.0	17.4±0.0	19.4±0.0	19.6±0.0	19.6±0.0	18.3±0.0	18.4±0.0	18.7±0.0	19±0.0	18.2±0.0	18.4±0.0	20.5±0.0	20±0.0	19.4±0.0	19.8±0.0	18.9±0.0	19.1±0.0	18.8±0.0	19.3±0.0	19.3±0.0	18.6±0.0	18.3±0.0
Q-3-rha	4.4±0.0	4.5±0.0	5.8±0.0	5.5±0.0	6.0±0.0	5.3±0.0	5.1±0.0	5.6±0.0	5.6±0.0	5.4±0.0	5.6±0.0	6.1±0.0	6.3±0.0	6.0±0.0	5.6±0.0	6.1±0.0	5.5±0.0	5.6±0.0	5.8±0.0	5.8±0.0	5.2±0.0	5.5±0.0
I-3-gal	1.1±0.0	1.8±0.0	1.7±0.0	1.7±0.0	1.3±0.0	1.7±0.0	1.6±0.0	1.7±0.0	1.7±0.0	1.5±0.0	1.7±0.0	1.8±0.0	1.8±0.0	2.6±0.0	1.6±0.0	1.9±0.0	1.6±0.0	1.6±0.0	1.7±0.0	1.7±0.0	1.7±0.0	1.5±0.0
S-3-gal	2.3±0.0	3.5±0.0	3.4±0.0	3.3±0.0	3.4±0.0	3.4±0.0	3.3±0.0	3.3±0.0	3.2±0.0	3.2±0.0	3.1±0.0	3.8±0.0	4.1±0.0	5.0±0.0	3.1±0.0	4.2±0.0	3.2±0.0	3.1±0.0	3.1±0.0	3.2±0.0	3.0±0.0	3.0±0.0
MQpent	3.8±0.0	5.7±0.0	5.7±0.0	5.6±0.0	5.5±0.0	5.3±0.0	5.5±0.0	5.4±0.0	5.3±0.0	5.2±0.0	5.3±0.0	5.3±0.0	5.7±0.0	6.8±0.0	5.2±0.0	5.6±0.0	5.3±0.0	5.4±0.0	5.4±0.0	5.2±0.0	5.1±0.0	5.2±0.0
I-3-ara	2.4±0.0	2.9±0.0	3.0±0.0	3.3±0.0	3.2±0.0	3.0±0.0	3.1±0.0	3.3±0.0	3.1±0.0	3.2±0.0	3.4±0.0	3.4±0.0	3.3±0.0	4.1±0.0	3.5±0.0	3.3±0.0	3.3±0.0	3.4±0.0	3.2±0.0	3.2±0.0	3±0.0	3.3±0.0
Q-3-pC-gal	0.9±0.0	1.6±0.0	1.4±0.0	1.3±0.0	1.4±0.0	1.4±0.0	2.1±0.0	1.8±0.0	1.4±0.0	1.7±0.0	1.9±0.0	3.6±0.0	3.2±0.0	2.9±0.0	2.4±0.0	2±0.0	2.4±0.0	2.1±0.0	2.1±0.0	1.6±0.0	1.7±0.0	2.1±0.0
pChex	2.8±0.0	4.5±0.0	4.5±0.0	4.6±0.0	4.2±0.0	4.3±0.0	2.9±0.0	4.4±0.0	4.3±0.0	4.3±0.0	4.1±0.0	4.0±0.0	4.3±0.0	4.5±0.0	4.3±0.0	4.2±0.0	4.6±0.0	4.7±0.0	4.4±0.0	4.4±0.0	4.2±0.0	4.4±0.0
Cdihex	1.8±0.0	3.4±0.0	3.2±0.0	3.3±0.0	3.0±0.0	3.0±0.0	10.4±0.0	3.1±0.0	2.9±0.0	2.9±0.0	2.6±0.0	2.6±0.0	3.2±0.0	3.3±0.0	3.3±0.0	3.1±0.0	3.3±0.0	3.4±0.0	3.4±0.0	3.4±0.0	3.3±0.0	3.4±0.0

Chex	7.2±0.0	11.0±0.0	10.8±0.0	10.9±0.0	10.6±0	10.5±0.0	1.9±0.0	10.9±0.0	10.5±0.0	10.5±0.0	10.2±0.0	10.2±0.0	11.0±0.0	11.0±0.0	11.0±0.0	10.5±0.0	11.1±0.0	11.0±0.0	11.0±0.0	10.9±0.0	10.6±0.0	10.6±0.0
Chexi	14.1±0.0	20.3±0.0	19.8±0.0	19.4±0.0	19.8±0	19.4±0	19.8±0	20.2±0	19.7±0	19.2±0	19.6±0	21±0	20.9±0	20.1±0	20.1±0	19.2±0	19.8±0	19.9±0	20.1±0	19.8±0	19.8±0	19.4±0
5CQ	101.0±0.2	146.2±0.3	143.1±0.3	143±0.3	141.9±0.3	137.9±0.3	140.2±0.3	143.7±0.3	138.3±0.3	137.2±0.3	136.8±0.3	148.5±0.3	149±0.3	145.9±0.3	146.1±0.3	139.8±0.3	146.7±0.3	145.2±0.3	146.6±0.3	144.9±0.3	142.3±0.3	140.4±0.3
pChexi	8.9±0.0	12.6±0.0	12.8±0.0	12.3±0.0	13±0	12.2±0	13±0	13.2±0	12.7±0	12.5±0	13.3±0	13.2±0	13±0	12.7±0	12.7±0	12.2±0	12.3±0	12.3±0	12.8±0	12.8±0	12.8±0	12.5±0
pChexi	14.5±0.0	21.1±0.0	20.4±0.0	20.5±0.0	20±0	19.6±0	19.6±0	20.1±0	19.5±0	19.2±0	18.9±0	21±0	21.2±0	20.7±0	20.8±0	19.8±0	21.1±0	20.9±0	20.9±0	20.6±0	20.4±0	20.1±0
Shex	10.8±0.0	15.9±0.0	16±0.0	16.2±0.0	16.6±0	15.2±0	16.3±0	18.2±0	16±0	15.9±0	16.3±0	25.1±0.1	16.7±0	18.5±0	16.8±0	16.8±0	15.7±0	16.3±0	16.6±0	16.3±0	16.4±0	16.2±0
APtet	8.6±0.0	12.5±0.0	12±0.0	10.4±0.0	12.1±0	11.5±0	12.1±0	12.5±0	12.1±0	11.8±0	11.9±0	13±0	12.8±0	12.8±0	12.6±0.0	11.7±0	12.4±0	12.6±0	12.4±0	12.5±0	12.3±0	12.2±0
Cat	10.3±0.0	15.2±0.0	15±0.0	12.3±0.0	14.8±0	14.5±0	14.6±0	15.1±0	14.5±0	14.3±0	14.2±0	14.8±0	15.3±0	15.2±0	15.3±0.0	2.9±0	3±0	2.9±0	3.1±0	15.1±0	14.9±0	14.6±0
BPdim	3.3±0.0	4.9±0.0	4.9±0.0	2.5±0.0	4.7±0	4.7±0	4.8±0	4.9±0	4.8±0	4.7±0	4.7±0	4.7±0	5.1±0	5.0±0.0	4.9±0.0	14.6±0	15.5±0	15.3±0	15.3±0	4.8±0	4.8±0	4.8±0
Epi	12.3±0.0	9.7±0.0	3.1±0.0	15.1±0.0	8.6±0	14.5±0	7.8±0	7.4±0	13.9±0	13.4±0	7.4±0	11.7±0	10.5±0	10.3±0	9.9±0.0	9.4±0	16.6±0	16.6±0	17.2±0	16.9±0	17.2±0	16.6±0
APtri	16.3±0.0	16.5±0.0	23.5±0.0	8.1±0.0	23.7±0	14.5±0	23.7±0	24.3±0	23.2±0	14.7±0	24±0	23.7±0	24.2±0	23.5±0.0	23.6±0.0	22.6±0	13.6±0	14.4±0	23.3±0	22.9±0	23±0	22.3±0
**SUM PC**	457.4±0.9	641.8±1.3	629.8±1.3	620.3±1.2	630.8±1.3	606.8±1.2	612.7±1.2	631.5±1.3	614.3±1.2	596±1.2	597.3±1.2	644.5±1.3	633.7±1.3	621.2±1.2	612.2±1.2	583.1±1.2	598.5±1.2	585.9±1.2	606.1±1.2	595±1.2	580.3±1.2	570.3±1.1

[1] Values are means ± standard deviation. n = 3;

Explanations:*- D-3-glu, Delfinidyn-3-*O*-glucoside; C-3-gal, Cyanidin-3-*O*-galactoside; C-3-glu, Cyanidin-3-*O*-glucoside; D-3-ara, Delphinidin-3-*O*-arabinoside; P-3-gal, Peonidin-3-*O*-galactoside; C-3-ara, Cyanidin-3-*O*-arabinoside; P-3-glu, Peonidin-3-*O*-glucoside; P-3-ara; Peonidin-3-*O*-arabinoside; M-3-gal, Malvidin-3-O-galactoside; M-3-ara, Malwidin-3-*O*-arabinoside; My-3-ara; Myricetin-3-O-arabinoside; My-3-gal, Myricetin-3-O-galactoside; My-3-glu, Myricetin-3-O-glucoside;

Q-3-gal, Quercetin-3-*O*-galactoside; Q-3-glu, Quercetin-3-*O*-glucoside; Q-3-rha, Quercetin-3-*O*-rhamnoside; I-3-gal, Isorhamnetin-3-*O*-galactoside; Q-3-pCQgal, Quercetin-3-*O*-(6″*p*-coumaroyl)-galactoside; pCQ, *p*-Coumaroyl-hexose isomer; Cdihex, Caffeoyl dihexoside; Chex, Caffeoyl hexoside; Chexi, Caffeoyl hexoside isomer; 5CQ, Chlorogenic acid; Shex, Sinapoyl-hexose; APtri, A-type PA-trimer; Cat, (+)-catechin; BPdim, B-type procyanidin-dimer; Epi, (-)-Epicatechin; APtet, A-type PA-tetramer.
